# Supplementary material for: The frequency response of networks as open systems
Source: Nat Commun. 2026 Jan 27;17:2088. doi: 10.1038/s41467-026-68602-1 (PMC12953583; doi:10.1038/s41467-026-68602-1)
Supplement: Supplementary file 1 — Supplementary Information [file 41467_2026_68602_MOESM1_ESM.pdf]

Supplementary Information for the paper:

The Frequency Response of Networks as Open Systems

Amirhossein Nazerian,[1] Malbor Asllani,[2] Melvyn Tyloo,[3,4] Wai Lim Ku,[5] and Francesco Sorrentino[1,6,a)]

[1)] *Department of Mechanical Engineering, University of New Mexico, Albuquerque, NM 87131, United States of America*

[2)] *Department of Mathematics, Florida State University, 1017 Academic Way, Tallahassee, FL 32306, United States of America*

[3)] *Living Systems Institute, University of Exeter, Exeter, EX4 4QD, United Kingdom*

[4)] *Department of Mathematics and Statistics, Faculty of Environment, Science, and Economy, University of Exeter, Exeter, EX4 4QD, United Kingdom*

[5)] *College of Medicine, Howard University, 2400 Sixth Street NW Washington, DC 20059, United States of America*

[6)] *Max Planck Institute for the Physics of Complex Systems, 01187 Dresden, Germany*

[a)] Electronic mail: fsorrent@unm.edu

Supplementary Note 1. CALCULATION DETAILS FOR THE DIRECTED CHAIN

A. Homogeneous self-feedback

Here we give a few calculation steps needed to obtain Eq. (9) in the main text. Using Eq. (8) in the main text, one has the $\mathcal{H}_2$-norm,

$$\|G\|_2^2 = \frac{1}{2\pi}\int_{-\infty}^{\infty} \frac{\xi_N^2}{(\omega + c\,J)^N(\omega - c\,J)^N}\,\mathrm{d}\omega\,. \tag{S1}$$

One can explicitly calculate the above integral by choosing e.g. a contour in the upper half of the complex plane, encircling the pole $z = c\,J$. Using the Residue theorem, one has,

$$\|G\|_2^2 = \frac{J}{(N-1)!}\lim_{\omega\to c\,J}\frac{\mathrm{d}^{N-1}}{\mathrm{d}\omega^{N-1}}\frac{\xi_N^2}{(\omega + c\,J)^N} \tag{S2}$$

$$= \frac{(-1)^{N-1}(2N-2)!}{(N-1)!(N-1)!}\frac{\xi_N^2}{(2c)^{2N-1}J^{2N-2}} \tag{S3}$$

$$= \frac{\binom{2N-2}{N-1}}{2^{2N-1}}\frac{\xi_N^2}{c^{2N-1}}\,. \tag{S4}$$

The last equality corresponds to the formula given in Eq. (9) of the main text.

B. Heterogeneous self-feedback

When the self-feedback c_i's are different at each node, one has the overall transfer function,

$$G(s) = \frac{w_1 w_2 \dots w_{N-1}}{(s+c_1)(s+c_2)...(s+c_N)} = \xi_N \prod_{i=1}^{N}\frac{1}{(s+c_i)}\,. \tag{S5}$$

The $\mathcal{H}_2$-norm then reads,

$$\|G\|_2^2 = \frac{\xi_N^2}{2\pi}\int_{-\infty}^{\infty}\prod_{i=1}^{N}\frac{1}{(\omega + c_i\,J)}\frac{1}{(\omega - c_i\,J)}\,\mathrm{d}\omega\,. \tag{S6}$$

Instead of two poles as in the homogeneous case, one has $2N$ poles given by $z_{i,\pm} = \pm c_i\,J$. Once again, using a contour integral in the upper half of the complex plane one has,

$$\|G\|_2^2 = (-1)^N\sum_{i=1}^{N}\frac{\xi_N^2}{2c_i}\prod_{j=1;j\neq i}^{N}\frac{1}{(c_i+c_j)}\frac{1}{(c_i-c_j)}\,. \tag{S7}$$

To make the dependence in the parameters more explicit, one can rewrite the latter equation as,

$$\|G\|_2^2 = (-1)^N \sum_{i=1}^{N} \frac{w_i^2}{2c_i} \prod_{j=1;j\neq i}^{N} \frac{w_j}{(c_i + c_j)} \frac{w_j}{(c_i - c_j)} \tag{S8}$$

$$= (-1)^N \xi_N^2 \sum_{i=1}^{N} \frac{1}{2c_i} \prod_{j=1;j\neq i}^{N} \frac{1}{(c_i^2 - c_j^2)} \,. \tag{S9}$$

Note that the above expression is valid no matter the signs of w_i's, c_i's, provided that the system is stable. Also, by combining the latter approach together with Supplementary Note 1 A, one can obtain an expression when some c_i's are the same.

Supplementary Note 2. NOISY INPUT SIGNALS

Sometimes, the network inputs are not known, but one still have some information about their distribution. In such a situation, one can evaluate some properties of the state distribution given the input random variables. We show here how the results presented in the main text about the trace of the Gramian relates to the case of noisy input signals. We consider the following linear dynamics,

$$\dot{\boldsymbol{x}}(t) = A\,\boldsymbol{x}(t) + B\,\boldsymbol{u}(t)\,, \tag{S10}$$

$$\dot{\boldsymbol{y}}(t) = C\,\boldsymbol{x}(t)\,, \tag{S11}$$

where we assume the matrix $A \in \mathbb{R}^{n\times n}$ is Hurwitz, i.e., its eigenvalues all have negative real part. The inputs are $\boldsymbol{u} \in \mathbb{R}^m$, and the matrix $B \in \mathbb{R}^{n\times m}$. The matrix C is the output matrix and the inputs $\boldsymbol{u}(t)$ are now stochastic and satisfy,

$$\mathbb{E}[u_i(t)] = \mu_i\,,\ \mathbb{E}[u_i(t)u_j(t')] = \delta_{ij}\,\nu_i^2\,\delta(t-t')\,. \tag{S12}$$

The latter means that the inputs are independent among different nodes, and uncorrelated in time. The variance of the inputs is then,

$$\mathrm{Var}[u_i(t)] = \nu_i^2 - \mu_i^2\,. \tag{S13}$$

Using this information about the input distribution, one can calculate some moments of the state. The expectation of the state is given by,

$$\mathbb{E}[\boldsymbol{y}(t)] = C\,A^{-1}(1 - e^{-A\,t})B\,\boldsymbol{\mu}\,, \tag{S14}$$

where A^{-1} stands for either the inverse or the pseudo-inverse of A. Therefore, in the long time limit, the mean value for the state is given by,

$$\lim_{t\to\infty} \mathbb{E}[\boldsymbol{y}(t)] = C\,A^{-1}B\boldsymbol{\mu}\,. \tag{S15}$$

The second moment is given by,

$$\mathbb{E}[\boldsymbol{y}(t)\boldsymbol{y}^\top(t)] = \int_0^t C\,e^{A\,(\tau-t)}B\,V\,B^\top e^{A^\top\,(\tau-t)}\,C^\top \mathrm{d}\tau\,, \tag{S16}$$

where we defined the diagonal matrix V whose components are given by $V_{ij} = \delta_{ij}\nu_i\nu_j$. One can include the heterogeneity of the inputs into a new matrix $\overline{B}_{ij} = B_{ij}\nu_j$. Doing so, one rewrites Eq. (S16) as,

$$\mathbb{E}[\boldsymbol{y}(t)\boldsymbol{y}^\top(t)] = \int_0^t C\,e^{A\,(\tau-t)}\overline{B}\,\overline{B}^\top e^{A^\top\,(\tau-t)}\,C^\top \mathrm{d}\tau = \overline{W}_c^{out}\,, \tag{S17}$$

which is simply the output Gramian matrix for an equivalent system. Without loss of generality, assuming that $\boldsymbol{\mu} = \boldsymbol{0}$, the total output variance is obtained by taking the trace of Eq. (S17) as,

$$\sum_{i=1}^{N} \mathbb{E}[y_i^2(t)] = \mathrm{Tr}[\overline{W}] = \sum_{i=1}^{N} \lambda_i(\overline{W}_c^{out})\,. \tag{S18}$$

As expected, one remarks that the output variance Eq. (S18) is the same expression as the $\mathcal{H}_2$-norm given in Eq. (9) in the main text[1–4]. To evaluate the impact of the noisy inputs one can consider the output standard deviation given by,

$$\sigma = \sqrt{\sum_{i=1}^{N} \mathbb{E}[y_i^2(t)]} = \sqrt{\sum_{i=1}^{N} \lambda_i(\overline{W}_c^{out})} = \sqrt{\|G\|_2^2} = \|G\|_2, \tag{S19}$$

where $\|G\|_2$ is the $\mathcal{H}_2$-norm of the transfer function $G(s) = C(sI - A)^{-1}B$. Similarly to the gain in the deterministic case, the output standard deviation is determined by the spectrum of the controllability Gramian, and consequently, the $\mathcal{H}_2$-norm.

Supplementary Note 3. CLOSED-FORM FORMULA OF BODE MAGNITUDE INTEGRAL

Consider the transfer function

$$G(s) = C(sI - A)^{-1}B \tag{S20}$$

where $A \in \mathbb{R}^{N\times N}$, $B = \boldsymbol{e}_i$, $C = \boldsymbol{e}_j^\top$, and $\boldsymbol{e}_k$ is canonical vector k. This suggests that node i is the input node and node j is the output node. We consider the case that the matrix A is possibly asymmetric, but diagonalizable with distinct eigenvalues, i.e., $A = V\Lambda V^{-1}$ where $V, \Lambda \in \mathbb{C}^{N\times N}$ and $\Lambda = \mathrm{diag}(\lambda_1, \lambda_2, \dots, \lambda_N)$, and $\lambda_i \neq \lambda_j$, $\forall i \neq j$. The matrix V has eigenvectors of the matrix A as its columns.

It follows

$$A^{-1} = V\Lambda^{-1}V^{-1}, \qquad (sI - A)^{-1} = -V(sI-\Lambda)^{-1}V^{-1}. \tag{S21}$$

By defining $W := V^{-1}$, we write

$$G(s) = C(sI-A)^{-1}B = -\sum_{k=1}^{N} \frac{V_{jk}W_{ki}}{s-\lambda_k} \tag{S22}$$

where $V_{jk}, W_{ki}, \lambda_k \in \mathbb{C}$ in general. We now study the magnitude of the frequency analysis, i.e., $|G(J\omega)|$ where $J = \sqrt{-1}$ is the imaginary unit.

The Bode magnitude integral in this case is

$$\int_0^\infty |G(J\omega)|^2 d\omega = -\pi \sum_{p=1}^{N}\sum_{q=1}^{N} \frac{V_{jp}W_{pi}\overline{V_{jq}}\,\overline{W_{qi}}}{\lambda_p + \overline{\lambda_q}} \tag{S23}$$

where $\overline{x}$ is the conjugate of x. In vector form, the above is written as

$$\int_0^\infty |G(J\omega)|^2 d\omega = \pi \left(V_{j,:}^\top \circ W_{:,i}\right)^* D \left(V_{j,:}^\top \circ W_{:,i}\right), \tag{S24}$$

where $D = [D_{pq}]$ and $D_{pq} = -1/(\lambda_p + \overline{\lambda_q})$, and * denotes conjugate transpose. The matrix D is Hermitian and positive semi-definite. The terms $V_{j,:}$ and $W_{:,i}$ are row j of the matrix V and column i of W, respectively.

Using the fact that the integrand is a quadratic form in the vector $\mathbf{a} = V_{j,:} \circ W_{:,i}^\top$, we can apply the inequality $|\mathbf{a}^\top M\mathbf{a}| \leq \|D\| \cdot \|\mathbf{a}\|^2$ and obtain the upper bound

$$\int_0^\infty |G(J\omega)|^2 d\omega \leq \pi\|D\| \cdot \|V_{j,:}\|^2 \cdot \|W_{:,i}\|^2. \tag{S25}$$

Since $\|V_{j,:}\| \leq \|V\|_F$ and $\|W_{:,i}\| \leq \|V^{-1}\|_F$, the bound becomes

$$\int_0^\infty |G(J\omega)|^2 d\omega \leq \pi\|D\| \cdot \kappa_F(V)^2, \tag{S26}$$

where $\kappa_F(V) = \|V\|_F \cdot \|V^{-1}\|_F$ serves as a measure of the non-normality of the matrix A. The Frobenius norm is defined as $\|V\|_F = \left(\sum_{i,j} |V_{ij}|^2\right)^{1/2}$, corresponding to the Euclidean

norm of the matrix entries treated as a vector. This shows that the original integral is directly influenced by the non-normality of the matrix A, as its magnitude is controlled by the condition number $\kappa_F(V)$. When A is far from normal, $\kappa_F(V)$ becomes large, leading to a potential amplification of the integral even if the eigenvalues themselves remain unchanged. In the special case where A is normal, V is unitary and the minimum possible value of the condition number is $\kappa_F(V) = \sqrt{N} \cdot \sqrt{N} = N$.

Supplementary Note 4. EMPIRICAL NETWORKS DATA

In this section, we provide complete information on empirical networks data.

A. Food webs

The next dataset is a food network with 36 nodes, known as Chesapeake[5]. The Total Dependency Coefficient (TDC) provides a fundamental measure of carbon transfer within the Chesapeake Bay food web, quantifying the percentage of carbon reaching a species that has previously passed through another species. This allows for identifying key energy pathways and dependencies among functional groups. The trophic structure of the Chesapeake ecosystem is established based on biomass measurements, which track the carbon flow between different species. At the base of the food web, phytoplankton and benthic diatoms serve as the primary producers, converting sunlight into organic carbon that fuels higher trophic levels. Additionally, dissolved organic carbon (DOC) and particulate organic carbon (POC)—both suspended (Suspended POC) and sedimented (Sediment POC)—act as essential energy sources for microbial and detrital pathways. These inputs sustain microbial decomposers, such as attached bacteria and sediment bacteria, as well as primary consumers, including microzooplankton, zooplankton, suspension feeders, and deposit feeders. As energy moves up the trophic chain, suspension-feeding fish and benthic-feeding fish feed on lower trophic levels, while carnivorous fish function as top predators, preying on other fish and larger consumers. By examining TDC values, we gain insight into how carbon propagates through the ecosystem, identifying critical species that facilitate energy flow and contribute to the stability and function of the Chesapeake Bay food web. Here, the matrix A has non-negative entries. To avoid numerical issues in calculating the continuous-time

controllability Gramian, we shifted the matrix $A \leftarrow A - cI$ such that the largest real part eigenvalue becomes -1. The matrix B is diagonal and has 5 nonzero entries on its main diagonal.

The last dataset is a food network with 65 nodes, known as Cypress Wetland[6]. Here, the matrix A has non-negative entries. To avoid numerical issues in calculating the continuous-time controllability Gramian, we shifted the matrix $A \leftarrow A - cI$ such that the largest real part eigenvalue becomes -1. The matrix B is diagonal and has 12 nonzero entries on its main diagonal.

B. Connectomes

The connectomes of Cat, Macaques 30, 32, 47, and 71 are structured into two main groups: areas that receive direct external input and those involved in higher-order processing. The external input areas are the brain's first stop for sensory information, receiving signals from the outside world via the thalamus or subcortical structures. The visual system processes input from the retina through the lateral geniculate nucleus (LGN), with V1 (primary visual cortex, 17) handling basic vision and V2 (18) refining it. Motion-sensitive areas like the lateral suprasylvian regions (PLLS, PMLS, AMLS, ALLS) integrate thalamic and subcortical motion signals. The auditory system follows a similar path, where AI (primary auditory cortex, 17) gets input from the cochlea via the medial geniculate nucleus (MGN), and AII, AAF, and P process additional sound cues. The somatosensory system, responsible for touch and proprioception, receives input via the thalamus (VPc) and distributes it to areas like 3a, 3b, 1, 2, and SII, each specializing in different aspects of body sensation (e.g., fine touch, deep pressure, proprioception). The higher-order processing areas do not receive raw sensory input but instead integrate, refine, and interpret information from primary sensory regions. Higher visual areas (V3/19, lateral suprasylvian cortex, and temporal visual association areas 21a and 21b) are responsible for object recognition, spatial awareness, and complex visual processing. Multisensory integration happens in regions like the posterior parietal cortex (7) and anterior ectosylvian sulcus (AES), where visual, auditory, and somatosensory information are combined. Motor-related regions (4g, 6l, 6m, 4) take in sensory and planning signals to guide movement execution. The prefrontal cortex (PFCMil, PFCMd, PFCL), limbic system (Cga, CGp, RS), and hippocampal-associated ar-

eas (perirhinal cortex, entorhinal cortex, subicular complex) play crucial roles in memory, decision-making, and spatial navigation. In short, sensory information first arrives in input regions before being progressively analyzed, integrated, and refined by higher-order areas, forming a structured network that enables perception, movement, and cognition.

C. Power grids

For the power grid datasets, the A matrix describes the dynamics of the voltage phases around the operational state. More precisely, it corresponds to the Jacobian of the linearized dynamics around the stable fixed point. The degree of freedom at each node is the phase of the complex voltage. The phase differences define the power flows over the transmission lines. Neglecting the losses on the transmission lines, the matrix A is symmetric. This is usually a good first approximation for high-voltage grids as the resistance is typically less than 10% of the reactance. Including the losses, the Jacobian matrix is not symmetric anymore. As we only consider high-voltage grids here, even when the losses are taken into account, the A matrix is close to be symmetric as demonstrated in Fig.3 of the main text. The IEEE 30-bus system has 6 synchronous generator and 41 transmission lines[7]. The IEEE 39-bus system has 10 synchronous generators and 46 transmission lines[8]. The UK transmission grid has 120 buses, including 17 generators. The A matrix is symmetric as dissipation is neglected. The RTS-96 test case is a power grid made of three areas[9]. It has 73 buses, including 33 generators. The matrix A is non-symmetric due to the dissipation on the lines. The matrix B has 33 non-vanishing diagonal entries corresponding to the generator buses. The IEEE-57 bus test case[10]. It has 12 generator and 42 load buses. The remaining buses are transformers. For this power grid, we took into account the dissipation on the lines, such that A is non-symmetric. Here, the matrix A has non-negative entries. To avoid numerical issues in calculating the continuous-time controllability Gramian, we shifted the matrix $A \leftarrow A - cI$ such that the largest real part eigenvalue becomes -1. The matrix B is diagonal and has 7 nonzero entries on its main diagonal which correspond to the active generators.

The third dataset IEEE-118 bus test case[11]. It has 19 generators, 35 synchronous condensers, 177 lines, 9 transformers, and 91 loads. Here, the matrix A has non-negative entries and is asymmetric as we include dissipation. To avoid numerical issues in calculating the

continuous-time controllability Gramian, we shifted the matrix $A \leftarrow A - cI$ such that the largest real part eigenvalue becomes -1. The matrix B is diagonal and has 39 nonzero entries on its main diagonal.

D. Genetic networks

The first dataset is the network "Human T-cell,"[12] which is created based on an experimental technique called CRISPR knockouts to identify the TF-gene networks for human primary CD4 T cells. Human primary CD4 T cells include a wide spectrum of T cells; therefore, several TFs are required to maintain the cell functions, which can be considered as the regulators receiving environmental signals. TBX21 is essential for maintaining effector $CD4^+$ T cells involved in combating intracellular pathogens. STAT1 is required to maintain $CD4^+$ T-cell responses to inflammatory signals. KMT2A is the upstream regulator of STAT5A/B, IRF4, and IL2RA, which are critical for $CD4^+$ T-cell survival and Treg function. RELA is essential for maintaining $CD4^+$ T-cell activation and responsiveness. DR1, YBX1, and BPTF is essential in maintaining $CD4^+$ T-cell transcription through unexpected, broad regulatory roles, highlighting the value of perturbation studies. The seven TFs described above are designated as input nodes.

The next dataset is "Stem cells 44", a TF-TF network of mouse embryonic stem cells[13]. Four transcription factors that regulate stemness are chosen as input nodes: POU5F1, SOX2, KLF4, and MYC.

The next dataset is a gene regulatory network of "Mouse Liver"[14]. The circadian clock, which is observed in the mouse liver, is regulated by the central circadian clock located in the suprachiasmatic nucleus (SCN) of the hypothalamus. The day-night cycle is the primary environmental cue that entrains the SCN of the hypothalamus. The SCN orchestrates systemic rhythms throughout the body by regulating hormonal secretion, body temperature, autonomic nervous system output, and feeding behavior. These systemic cues serve as time-giving signals that synchronize the peripheral clocks in the liver, which are maintained by the core clock genes, including Clock, Nr1d1, and Rora. The three TFs (Clock, Nr1d1, and Rora) are related to central circadian clock and considered as input nodes.

E. Pathway networks

Pathway maps in the KEGG database[15] are graphical summaries of how biological molecules interact. Each map is drawn as a network in which nodes stand for concrete molecular entities—genes or proteins, small metabolites, protein complexes, or second messengers such as cAMP or Ca^{2+}. Edges show the type of relationship between these entities, for example, activation, inhibition, transport, or binding. In this way, a pathway network offers a compact view of the information and material flow that underlies a cellular response. The input nodes are usually extracellular ligands, membrane-spanning receptors, or transport proteins embedded in the plasma membrane. Classic signaling cascades (e.g., MAPK, PI3K-Akt, or calcium signaling) begin with an extracellular ligand binding to a cell-surface receptor. Both the soluble ligand and its transmembrane receptor qualify as input nodes: perturbing either one alters the downstream phosphorylation events, second-messenger bursts, and transcriptional changes depicted deeper in the map. The input nodes are identified by scanning for (i) extracellular ligands positioned at the edge of the map, (ii) membrane-spanning proteins that bind or transport those ligands, and (iii) specialized sensor proteins that initiate internal signaling upon detecting environmental cues.

A summary of the information on all real networks considered in this study is provided in Table I.

Supplementary Note 5. REAL NETWORKS ANALYSIS BASED ON THE NETWORK ADJACENCY MATRIX

For many empirical networks, the adjacency matrix $\tilde{A}$ is readily available, whereas determining the network Jacobian typically requires additional modeling assumptions about the underlying dynamics and the choice of a fixed point, which may not be unique. To circumvent these limitations, in this note we replace the network Jacobian with a shifted adjacency matrix. Specifically, we apply the transformation $A \leftarrow A - cI$ and choose the parameter c so that the largest real-part eigenvalue of the shifted A is equal to -1. As we show below, this approximation allows us to reproduce the results in Figs. 3 and 4 of the main manuscript and yields outcomes that are remarkably consistent with those obtained using the full Jacobian-based formulation.

Table I: Real networks information.

Category	Networks	Nodes	Edges	Num. of inputs	Node info	Edge info	weighting	Ref.
Gene networks	Stem cells 44	44	547	4	Transcription factors (TF) & target genes (TG)	Molecular interaction	unweighted	[13]
	Human Tcell	47	227	7			unweighted	[12]
	Mouse liver	210	1910	3			unweighted	[14]
	net_p_aeruginosa	648	959	33			unweighted	[16]
	net_yeast	662	1063	86			unweighted	[16]
	Gene_545	545	980	527			unweighted	[17]
Power grids	IEEE 30	30	82	6	Generators (power generation) or loads (power consumption)	Transmission lines	weighted	[7]
	IEEE 39	39	92	10			weighted	[8]
	IEEE 57	57	156	7			weighted	[10]
	RTS96	73	216	33			weighted	[9]
	IEEE 118	118	358	39			weighted	[11]
	UK grid	120	330	17			weighted	[18,19]
Food web	Blackrocktxt	86	375	49	Resource species (prey) & consumer species (predator)	Consumption relation	unweighted	[16]
	Broadtxt	94	564	53			unweighted	[16]
	BurgessShaleS10b_w	48	243	6			weighted	[20]
	DempstersAutxt	83	414	46			unweighted	[16]
	Germantxt	84	352	48			unweighted	[16]
	Kyeburntxt	98	629	58			unweighted	[16]
	LilKyeburntxt	78	375	42			unweighted	[16]
	NorthColtxt	78	241	25			unweighted	[16]
Connectome	C-elegans	283	4690	86	Neurons or brain regions	Neural connections	weighted	[21]
	Cat	52	818	16			weighted	[22]
	Macaque 30	30	311	7			unweighted	[23]
	Macaque 32	32	315	7			unweighted	[23]
	Macaque 47	47	505	10			unweighted	[24]
	Macaque 71	71	746	16			unweighted	[25]
	Mouse brain	213	21654	34			unweighted	[26]
Pathway	hsa04014	56	65	21	Gene product, mostly protein but including RNA	Molecular interaction or relation	unweighted	[15]
	hsa04022	60	64	18			unweighted	[15]
	hsa04066	33	34	4			unweighted	[15]
	hsa04071	28	36	7			unweighted	[15]
	hsa04150	48	52	18			unweighted	[15]
	hsa04151	64	67	29			unweighted	[15]
	hsa04152	45	44	24			unweighted	[15]
	sce04011	24	27	5			unweighted	[15]

By using the analytical tools introduced in this paper, particularly the $\mathcal{H}_2$-norm, we now analyze the blocking versus passing behavior of several empirical networks. As already mentioned, in this section, we take the matrix A to coincide with the shifted adjacency matrix of each network. All the adjacency matrices that we consider here are either unweighted or weighted with non-negative weights (see Supplementary Note 4). We then use the pair (A, B) to calculate the trace of the Controllability Gramian $\mathrm{Tr}(W_c)$ and the $\mathcal{H}_2$-norm. For each dataset, we fix the number of input nodes m to its empirical (measured) value. We first choose uniformly at random 10,000 sets of m nodes without repetitions, set them as input nodes, and evaluate $\mathrm{Tr}(W_c)$, the trace of the infinite-horizon continuous-time controllability Gramian. We compare the resulting $\mathrm{Tr}(W_c)$ from randomly chosen input nodes with the $\mathrm{Tr}(W_c)$ resulting from input nodes provided by the empirical data. The comparison is performed by evaluating two measures:

$$z\text{-score} = \frac{x - \bar{x}}{s}, \quad p\text{-value} = \int_{x_{\min}}^{x_{real}} p(x)dx \mod 0.5, \tag{S27}$$

where $\bar{x}$ is the mean of the sample, s is the standard deviation of the sample, $x_{\min}$ is the minimum of the sample, x_{real} is the real data choice, and $p(x)$ is the probability density function such that $\int_{x_{\min}}^{x_{\max}} p(x)dx = 1$. We define

$$\text{Passing network}: \begin{cases} \text{Low } p\text{-value}, \\ \\ \text{large positive } z\text{-score}, \end{cases}$$

$$\text{Blocking network}: \begin{cases} \text{Low } p\text{-value}, \\ \\ \text{large negative } z\text{-score}, \end{cases}$$

and we recall that extreme (large magnitude) z-scores correspond to low p-values, indicating statistically *atypical* configurations, while small z-scores and high p-values reflect *typical* behavior. The results of z-score vs p-value are shown in Supplementary Fig. 1 a. We consider different categories of networks: power grids for which input nodes are generators, connectomes for which input nodes are sensory neurons, molecular signaling networks for which input nodes are receptors, gene regulatory networks for which input nodes are transcription factors, pathway networks for which input nodes are extracellular ligands, and food

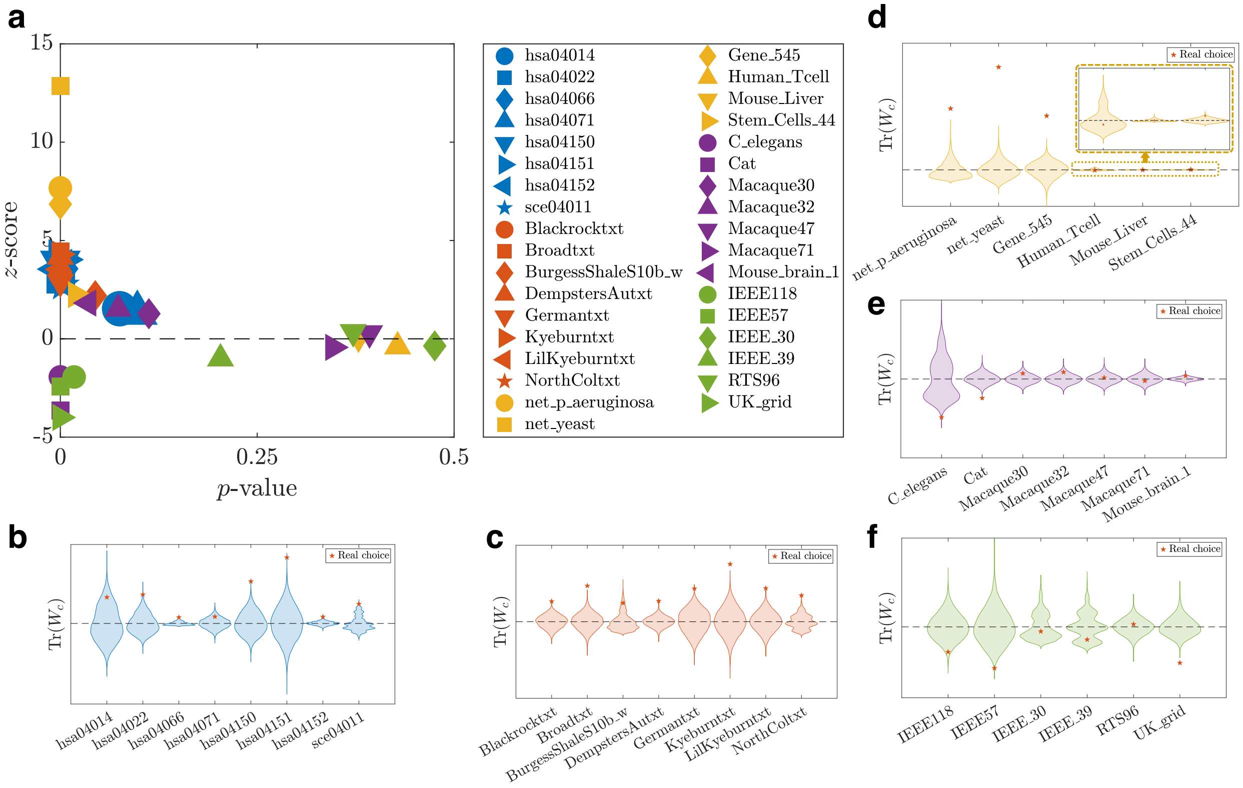

Supplementary Fig. 1: **Empirical data analysis.** Panel a shows the z-score vs p-value of the real data choice of the input nodes based on evaluation of the trace $\mathrm{Tr}(W_c)$ of real network data (based on their adjacency matrix A and the input matrix B). Panels b-f show the distributions of $\mathrm{Tr}(W_c)$ for selected real networks over 10,000 sets of randomly chosen input nodes. The number of randomly input nodes is the same as the number of input nodes from the real data. For each network, the choice of real input nodes is plotted as a red star. The violin plots are shifted for better visualization, such that the mean of each distribution lies on the dashed black line. Networks within the same family are plotted using the same color in different panels: b: Pathway networks (blue), c: Food Webs (orange), d: Genetic network (yellow), e: Connectomes (purple), and f: Power Grids (green).

webs for which input nodes are autotrophs (plants and algae) that produce their food via photosynthesis.

Supplementary Fig. 1 shows that in pathway networks and food webs, the real choice of input nodes results in passing behavior, while power grids are either blocking or typical. Genetic networks are either passing or typical, and connectomes do not show a discernible

pattern since some networks are passing, some are blocking, and the rest are typical. The violin plots confirm our conclusions, while providing a more detailed view, as they display the entire distribution: atypical values appear in the tails, whereas typical values cluster near the peak, usually centered around the mean.

Supplementary Fig. 2 a and b show the normalized trace of the controllability Gramian, $\mathrm{Tr}(W_c)/N^2$, and the trace of the controllability Gramian, $\mathrm{Tr}(W_c)$, versus the Henrici index of the adjacency matrix $\hat{d}_F(A)$, respectively. We observe a clear trend: higher Henrici index correlates with higher normalized trace and higher trace. Moreover, networks in the same category cluster together in this plot, revealing distinct levels of non-normality and signal amplification across categories. Notably, food webs, signaling pathways, and gene regulatory networks exhibit a pronounced scale separation—one to two orders of magnitude higher in normalized trace compared to other networks. This is consistent with their (almost) perfect directed acyclic graph (DAG) structure, which maximizes passing behavior along input–output paths. Interestingly, while food webs and pathways achieve this via fewer but longer paths, gene regulatory networks exhibit many very short paths, resembling a collection of directed star graphs[27]. As further detailed in the Supplementary Note 7, the unnormalized $\mathrm{Tr}(W_c)$ reinforces this observation by clustering gene regulatory networks closer to food webs and pathways, underscoring the role of perfect directedness in enhancing the passing property.

In summary, the results obtained in this section closely mirror those presented in Figs. 3 and 4 of the main manuscript, confirming that the shifted-adjacency approximation faithfully captures the relevant network dynamics.

Supplementary Note 6. NETWORK GAIN

We consider a linear system with n degrees of freedom whose state is noted $\boldsymbol{x} \in \mathbb{R}^n$ and which is subject to bounded inputs. Its dynamics is governed by the set of coupled differential equations,

$$\dot{\boldsymbol{x}}(t) = A\,\boldsymbol{x}(t) + B\,\boldsymbol{u}(t) \tag{S28}$$

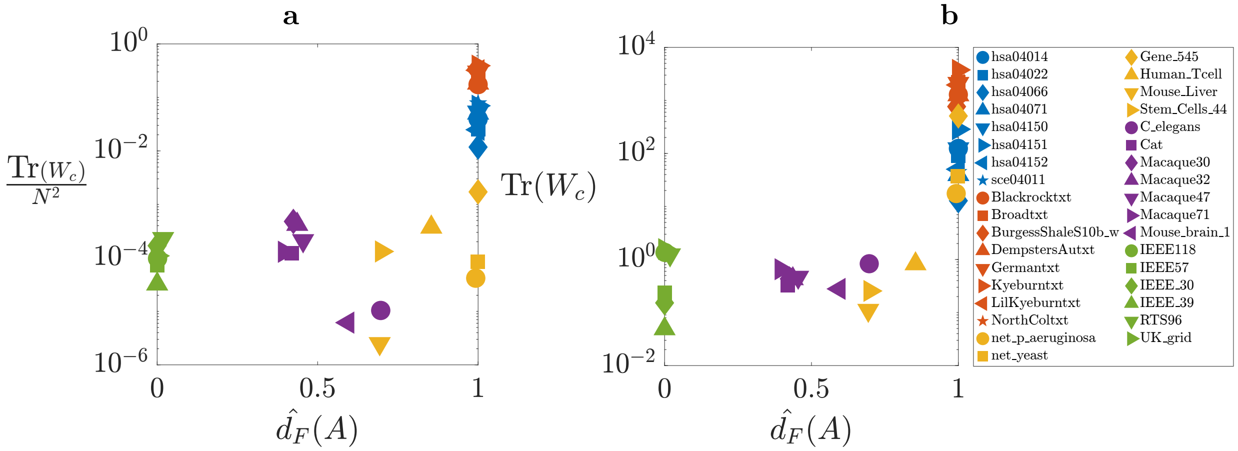

Supplementary Fig. 2: **a) The normalized trace of Gramian vs. the non-normality index, and b) The trace of Gramian vs. the non-normality index.** The normalized Henrici index $\hat{d_F}(A)$ is defined in the main manuscript. Pathway, Food web, Genetic, Connectome, and Power Grid networks are in blue, orange, yellow, purple, and green, respectively.

where in this section, similar to Supplementary Note 5, we take A to be the shifted adjacency matrix in place of the network Jacobian. The general solution of Eq. (S28) reads,

$$\boldsymbol{x}(t) = e^{-A\,t}\boldsymbol{x}_0 + e^{-A\,t}\int_0^t e^{A\,\tau} B\,\boldsymbol{u}(\tau)\,\mathrm{d}\tau\,. \tag{S29}$$

For a sufficiently long time, the first term on the right-hand side of the latter equation corresponding to the free evolution vanishes.

Eventually, we want to disentangle the relation between the parameters of the network and its response to a sequence of inputs. With this knowledge, one could then design networks that would maximize or minimize the response for a particular input sequence. More precisely, we evaluate the amplitude of $\boldsymbol{x}(t)$ given a sequence of inputs $\boldsymbol{u}(t')\,,\ t' < t\,$. In the following sections, we consider first deterministic and then stochastic inputs. We assume that we observe the system at a time t long enough such that the free evolution can be neglected and the state is

$$\boldsymbol{x}(t) = e^{-A\,t}\int_0^t e^{A\,\tau} B\,\boldsymbol{u}(\tau)\,\mathrm{d}\tau\,. \tag{S30}$$

We assume the pair (A, B) is controllable. We assume the initial condition of the system at time $t = 0$ is the origin, i.e., $\boldsymbol{x}(0) = \boldsymbol{0}$. It is known that the minimum energy required to

derive the system to the desired position $\boldsymbol{x}_d$ in time t is

$$E(\boldsymbol{x}_d, t) = \boldsymbol{x}_d^\top W_c^{-1}(t)\boldsymbol{x}_d \tag{S31}$$

where the symmetric positive definite matrix $W_c(t) = \int_0^t e^{A\tau}BB^\top e^{A^\top\tau}d\tau$ is the continuous-time controllability Gramian. We define the continuous-time network gain as the ratio of the farthest final position and the minimum energy required as

$$d_c(t) := \sup_{\boldsymbol{x}_d} \frac{\boldsymbol{x}_d^\top \boldsymbol{x}_d}{E(\boldsymbol{x}_d, t)} = \sup_{\boldsymbol{x}_d} \frac{\boldsymbol{x}_d^\top \boldsymbol{x}_d}{\boldsymbol{x}_d^\top W_c^{-1}(t)\boldsymbol{x}_d} \overset{\boldsymbol{y} := W_c^{-\frac{1}{2}}\boldsymbol{x}_d}{=} \sup_{\boldsymbol{y}} \frac{\boldsymbol{y}^\top W_c(t)\boldsymbol{y}}{\boldsymbol{y}^\top \boldsymbol{y}} = \lambda_{\max}(W_c(t)). \tag{S32}$$

In the case that the measurable outputs of the system at time t are $\boldsymbol{y}(t) = C\boldsymbol{x}(t) \in \mathbb{R}^q$, the minimum energy required to drive the outputs to the desired output $\boldsymbol{y}_d$ at time t is

$$E(\boldsymbol{y}_d, t) = \boldsymbol{y}_d^\top W_c^{out^{-1}}(t)\boldsymbol{y}_d, \tag{S33}$$

where the output controllability Gramian is $W_c^{out} = CW_cC^\top$. In this case, the gain becomes

$$d_c^{out}(t) = \lambda_{\max}(W_c^{out}(t)). \tag{S34}$$

Next, we study the level of blocking versus passing behavior of several empirical networks similar to the main manuscript, but using the metric $\lambda_{\max}(W_c)$ instead. For each dataset, we fix the number of input nodes m to its empirical value. We shift the adjacency matrices such that their largest real part eigenvalue is -1. We first randomly choose 10,000 sets of m nodes (with a uniform probability of selection) and set them as input nodes and evaluate $\lambda_{\max}(W_c)$, the largest eigenvalue of the infinite-horizon continuous-time controllability Gramian. We compare the resulting $\lambda_{\max}(W_c)$ from randomly choosing input nodes with the $\lambda_{\max}(W_c)$ resulting from input nodes of the empirical data. The comparison is performed by evaluating two measures: z-score and p-value, with the same definitions as in the main manuscript. The results of z-score vs p-value are shown in Supplementary Fig. 3 a. We considered different categories of networks: power grids for which input nodes are generators, connectomes for which input nodes are sensory neurons, molecular signaling networks for which input nodes are receptors, gene regulatory networks for which input nodes are transcription factors, and food webs for which input nodes are autotrophs (plants and algae) that produce their food via photosynthesis. For all networks, in the absence of more detailed information, we set $C = I$. The plots in which we show the distribution of $\lambda_{\max}(W_c)$ along with the real network choice are shown in Supplementary Fig. 3 b-c. We see that for Pathway networks, Food webs,

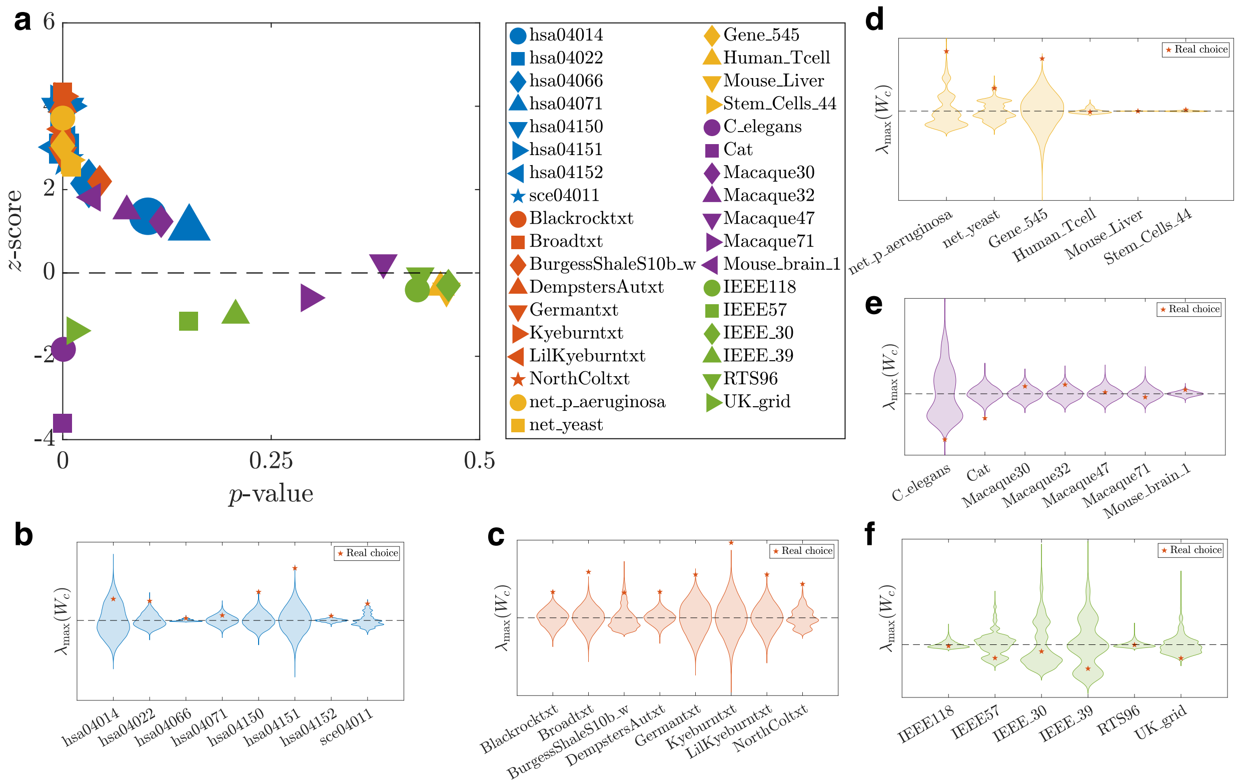

Supplementary Fig. 3: **Empirical data analysis: largest eigenvalue of continuous-time controllability Gramian.** Panel a shows the z-score vs p-value of the real data choice of the input nodes based on evaluation of $\lambda_{\max}(W_c)$ of real network data. Panels b-f show the distributions of $\lambda_{\max}(W_c)$ for selected real networks over 10,000 sets of randomly chosen input nodes. The number of randomly input nodes is the same as the number of input nodes based on the real data. For each network, the choice of real data input nodes is also plotted as a red star. The violin plots are shifted for better visualization, such that the mean of each distribution lies on the dashed black line. Networks within the same family are plotted using the same color in different panels: b: Pathway networks (blue), c: Food Webs (orange), d: Genetic network (yellow), e: Connectomes (purple), and f: Power Grids (green).

and Genetic networks, the real data choice of the input nodes results in a passing behavior. Power Grids tend to show a mix of blocking and typical behavior, while Connectomes do not show a discernible pattern since some networks are passing, some are blocking, and the rest are typical.

Supplementary Note 7. $\mathcal{H}_2$-NORM FOR EMPIRICAL NETWORKS

Supplementary Fig. 4 extends our disturbance-amplification analysis of real-world networks. In this section, similar to Supplementary Note 5, we take A to be the shifted adjacency matrix in place of the network Jacobian. For each data set, the spectrum of the adjacency matrix is shifted so that the dominant eigenvalue satisfies $\Re(\lambda_{\max}) = -1$, ensuring a common stability margin across all cases. We then enumerate every reachable input–output pair[28] and compute the corresponding $\mathcal{H}_2$-norm of the single-input/single-output (SISO) system $(A, B = e_i, C = e_j^\top)$. The resulting collection of $\mathcal{H}_2$ values is displayed as a log–log histogram (logarithmic axes for both frequency and norm). The power grids (UK Grid, RTS96, IEEE57, IEEE118) cluster tightly toward the lower end of the norm axis (left-hand side of their respective plots), with most mass between 10^{-30} and 10^{-2}. This confirms that, independent of the particular generator–load pairing, power-grid topologies are generally dissipative with respect to exogenous disturbances. The Connectomes (Cat, Mouse Brain, Macaque 47, 71, 30, 32) display norms two to six orders of magnitude larger than those of the grids. Their histograms are also broader and more bell-shaped, indicating a wider variability in disturbance amplification across different stimulus–response pairs. The Mouse Brain network exhibits the widest spread, reflecting its highly heterogeneous modular structure.

Supplementary Fig. 5 summarizes the disturbance-response characteristics of real-world networks by plotting the empirical distribution of their $\mathcal{H}_2$-norms under randomized input placements. For each data set, we first spectrally shift the adjacency matrix so that its dominant eigenvalue has real part -1, guaranteeing a uniform stability margin. We then fix the number of control inputs to the value reported with the data set (m) and, keeping the output matrix at $C = I_n$, draw 10^4 distinct input sets of size m uniformly at random from the n nodes. The resulting 10^4 $\mathcal{H}_2$-norm values are binned and displayed on a log–linear histogram (logarithmic frequency axis, linear norm axis); the red vertical line marks the $\mathcal{H}_2$ obtained with the "true" input configuration that accompanies the data set. The p-value printed in each panel is the empirical probability that a random input placement yields an $\mathcal{H}_2$-norm not larger than the real network's value. In the case of power-grid models (UK Grid, IEEE57, IEEE118, RTS96), all four grids exhibit comparatively small $\mathcal{H}_2$-norms: their red lines lie on the extreme left of the corresponding histograms, with p-values between 0

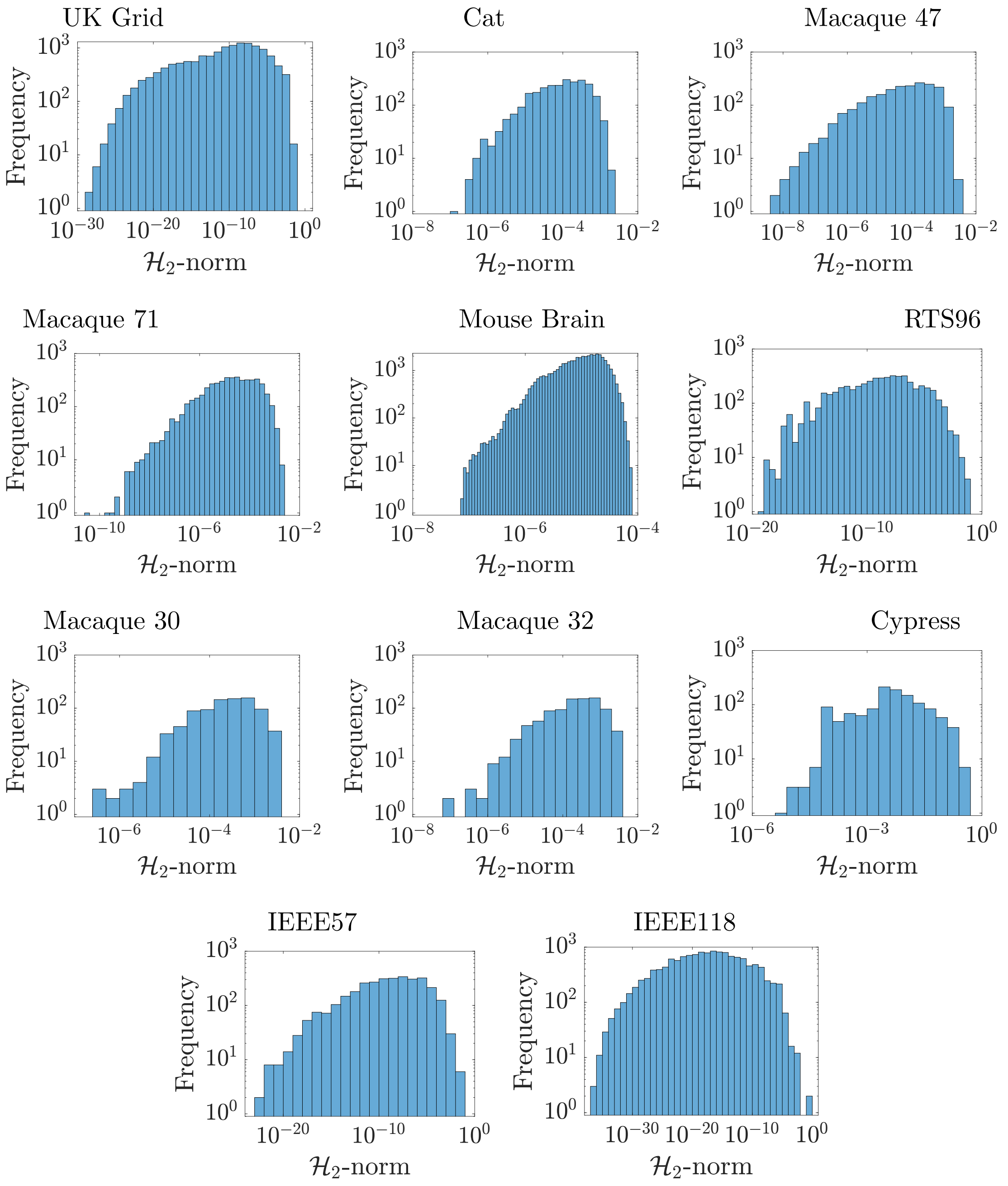

Supplementary Fig. 4: The histogram of the $\mathcal{H}_2$-norm for selected real datasets. For all data sets, all eigenvalues of the adjacency matrix are shifted such that the largest real part is -1. For each dataset, we evaluate the $\mathcal{H}_2$-norm for all possible reachable pairs of the input/output nodes. The red line shows the real data choice of input nodes with all nodes as the output nodes.

and 0.35. This indicates that the empirical placements of generator are unusually effective at damping network-wide disturbances relative to random choices. In the case of the Cat and macaque connectomes, the five neural connectomes (Cat, Macaque 47, 71, 30, 32) show markedly larger norms, with distributions centered farther to the right and empirical norms landing close to the modal region (p between 0.07 and 0.37). In other words, typical random input locations in these cortical graphs perform about as well as the biologically observed ones, suggesting that noise amplification is an inherent feature of the underlying topology rather than of input selection.

REFERENCES

[1] J. Doyle, K. Glover, P. Khargonekar, and B. Francis, IEEE Transactions on Automatic Control **34**, 831 (1989).

[2] K. Zhou and J. Doyle, *Essentials of Robust Control* (Prentice-Hall, Upper Saddle River, NJ, USA, 1998).

[3] M. R. Jovanović and M. Fardad, Automatica **44**, 2090 (2008).

[4] B. Hassibi, A. H. Sayed, and T. Kailath, *Indefinite-Quadratic Estimation and Control* (Society for Industrial and Applied Mathematics, Philadelphia, PA, USA, 1999).

[5] D. Baird and R. E. Ulanowicz, Ecological monographs **59**, 329 (1989).

[6] A. E. Krause, K. A. Frank, D. M. Mason, R. E. Ulanowicz, and W. W. Taylor, Nature **426**, 282 (2003).

[7] IEEE 30-bus test case, `http://www.ee.washington.edu/research/pstca/pf30/pg_tca30bus.htm` (Accessed: 2025-07-02).

[8] T. Athay, R. Podmore, and S. Virmani, IEEE Transactions on Power Apparatus and Systems , 573 (1979).

[9] C. Grigg, P. Wong, P. Albrecht, R. Allan, M. Bhavaraju, R. Billinton, Q. Chen, C. Fong, S. Haddad, S. Kuruganty, *et al.*, IEEE Transactions on power systems **14**, 1010 (1999).

[10] IEEE 57-bus test case, `http://www.ee.washington.edu/research/pstca/\pf57/pg_tca57bus.htm` (Accessed: 2024-03-08).

[11] R. Christie, IEEE 118 Tescase, `http://labs.ece.uw.edu/pstca/pf118/pg_tca118bus.htm` (1993).

[12] J. S. Weinstock, M. M. Arce, J. W. Freimer, M. Ota, A. Marson, A. Battle, and J. K.

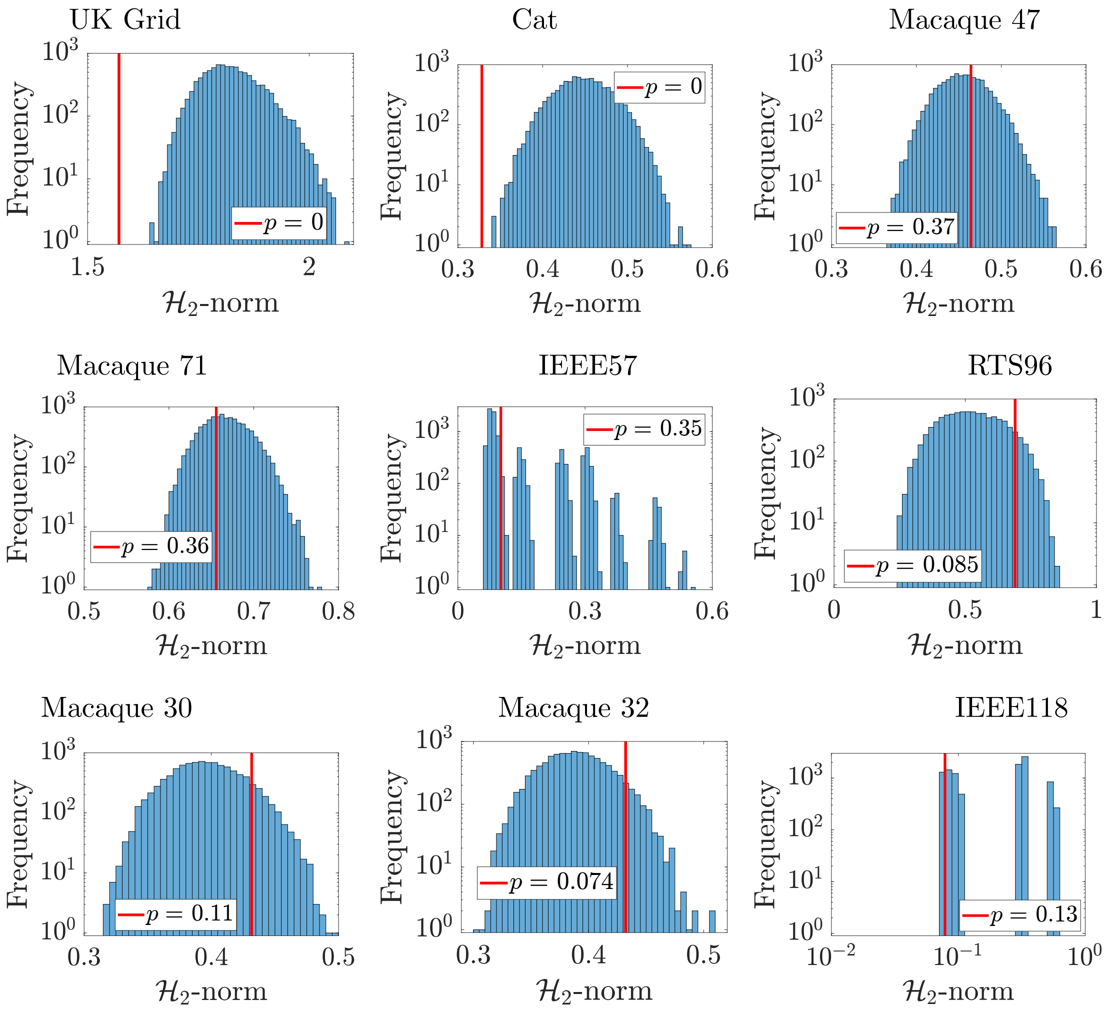

Supplementary Fig. 5: The histogram of the $\mathcal{H}_2$-norm for selected real datasets. For all data sets, all eigenvalues of the adjacency matrix are shifted such that the largest real part is -1. For each dataset, we evaluate the $\mathcal{H}_2$-norm for 10,000 randomly chosen m number of input nodes and select all nodes to be output nodes ($C = I$). The value m is the number of input nodes from the data set, and the real data is shown as the red line. The p-value corresponding to each dataset is shown as legend p.

Pritchard, Cell Genomics **4** (2024).

[13] H. Xu, C. Baroukh, R. Dannenfelser, E. Y. Chen, C. M. Tan, Y. Kou, Y. E. Kim, I. R. Lemischka, and A. Ma'ayan, Database **2013**, bat045 (2013).

[14] L. Fang, Y. Li, L. Ma, Q. Xu, F. Tan, and G. Chen, Nucleic acids research **49**, D97 (2021).

[15] K. Laboratories, Kegg pathway database (2025).

[16] S. Johnson and N. S. Jones, Proceedings of the National Academy of Sciences **114**, 5618

(2017).

[17]P. Barah, N. D. Jayavelu, R. Sowdhamini, K. Shameer, and A. M. Bones, Nucleic acids research **44**, 3147 (2016).

[18]I. Simonsen, L. Buzna, K. Peters, S. Bornholdt, and D. Helbing, Physical review letters **100**, 218701 (2008).

[19]R. Delabays, A. Y. Lokhov, M. Tyloo, and M. Vuffray, PRX Energy **2**, 023009 (2023).

[20]J. A. Dunne, R. J. Williams, N. D. Martinez, R. A. Wood, and D. H. Erwin, PLoS biology **6**, e102 (2008).

[21]L. R. Varshney, B. L. Chen, E. Paniagua, D. H. Hall, and D. B. Chklovskii, PLoS computational biology **7**, e1001066 (2011).

[22]J. W. Scannell, G. Burns, C. C. Hilgetag, M. A. O'Neil, and M. P. Young, Cerebral Cortex **9**, 277 (1999).

[23]D. J. Felleman and D. C. Van Essen, Cerebral cortex (New York, NY: 1991) **1**, 1 (1991).

[24]C. J. Honey, R. Kötter, M. Breakspear, and O. Sporns, Proceedings of the National Academy of Sciences **104**, 10240 (2007).

[25]M. P. Young, Proceedings of the Royal Society of London. Series B: Biological Sciences **252**, 13 (1993).

[26]S. W. Oh, J. A. Harris, L. Ng, B. Winslow, N. Cain, S. Mihalas, Q. Wang, C. Lau, L. Kuan, A. M. Henry, *et al.*, Nature **508**, 207 (2014).

[27]J. D. O'Brien, K. A. Oliveira, J. P. Gleeson, and M. Asllani, Physical Review Research **3**, 023117 (2021).

[28]For a given ordered pair (i, j), node i is treated as the single control input and node j as the single measured output; pairs that are not mutually reachable through the directed graph are discarded.
